# A Prospective Study of Bone Marrow Hematopoietic and Mesenchymal Stem Cells in Type 1 Gaucher Disease Patients

**DOI:** 10.1371/journal.pone.0069293

**Published:** 2013-07-25

**Authors:** Séverine Lecourt, Enguerran Mouly, Delphine Freida, Audrey Cras, Raphaël Ceccaldi, Djazia Heraoui, Christine Chomienne, Jean-Pierre Marolleau, Bertrand Arnulf, Raphael Porcher, Catherine Caillaud, Valérie Vanneaux, Nadia Belmatoug, Jérôme Larghero

**Affiliations:** 1 Assistance Publique-Hôpitaux de Paris, Hôpital Saint Louis, Unité de Thérapie Cellulaire et Centre d'Investigation Clinique en Biothérapies CIC-BT501, Paris, France; 2 INSERM UMRS940, Institut Universitaire d'Hématologie, Hôpital Saint-Louis, Paris, France; 3 EA3963, Univ Paris Diderot, Sorbonne Paris Cité, Institut Universitaire d'Hématologie, Paris, France; 4 INSERM U944, Institut Universitaire d'Hématologie, Hôpital Saint-Louis, Paris, France; 5 Assistance Publique-Hôpitaux de Paris, Hôpital Beaujon, Service de Médecine Interne, Centre de Réference pour les Maladies Lysosomales, Clichy, France; 6 Hôpital d'Amiens, Département d'Hématologie Clinique, Amiens, France; 7 Assistance Publique-Hôpitaux de Paris, Hôpital Saint Louis, Département d'Immuno-hématologie, Hôpital Saint-Louis, Paris, France; 8 Assistance Publique-Hôpitaux de Paris, Hôpital Saint Louis, Département de Biostatistiques et Information Médicale, Paris, France; 9 Laboratoire de Génétique Métabolique, Hopital Cochin, Paris, France; 10 Univ Paris Diderot, Sorbonne Paris Cité, Paris, France; University Hospital S. Maria della Misericordia, Udine, Italy

## Abstract

Gaucher disease (GD) is an autosomal recessive disorder characterized by lysosomal glucocerebrosidase (GBA) deficiency leading to hematological and skeletal manifestations. Mechanisms underlying these symptoms have not yet been elucidated. *In vivo*, bone marrow (BM) mesenchymal stem cells (MSCs) have important role in the regulation of bone mass and in the support of hematopoiesis, thus representing potential candidate that could contribute to the disease. GBA deficiency may also directly impair hematopoietic stem/progenitors cells (HSPCs) intrinsic function and induce hematological defect. In order to evaluate the role of BM stem cells in GD pathophysiology, we prospectively analyzed BM-MSCs and HSPCs properties in a series of 10 patients with type 1 GD. GBA activity was decreased in all tested cell subtypes. GD-MSCs had an impaired growth potential, morphological and cell cycle abnormalities, decreased capacities to differentiate into osteoblasts. Moreover, GD-MSCs secreted soluble factors that stimulated osteoclasts resorbing activities. *In vitro* and *in vivo* primitive and mature hematopoiesis were similar between patients and controls. However, GD-MSCs had a lower hematopoietic supportive capacity than those from healthy donors. These data suggest that BM microenvironment is altered in GD and that MSCs are key components of the manifestations observed in GD.

## Introduction

Gaucher Disease (GD) is a lysosomal storage disorder due to glucocerebrosidase (GBA, acid-β-glucosidase) deficiency [1]. Three clinical phenotypes are described based on the presence and degree of neurological involvement [2], [3]. In type 1 GD, the accumulation of glucosylceramide in the lysosomal compartment of affected cells results in heterogeneous manifestations including visceral, hematological and skeletal involvement [2].

Skeletal manifestations of GBA deficiency mainly consist in Erlenmeyer flask deformity, bone marrow (BM) infiltration, osteopenia, avascular necrosis, infarction, fractures, lytic lesions and joint replacements. Pathophysiology of these manifestations remains poorly understood and hypotheses include inhibition of osteogenesis and/or stimulation of osteoclastogenesis by cytokines. Plasma levels of soluble factors involved in the regulation of osteoblasts and osteoclasts activity have thus been shown to be deregulated in GD, but the results reported in several studies were inconstant and not correlated with clinical severity [3], [4], [5], [6], [7], [8]. Hematological consequences of GBA deficiency mainly consists in anemia and thrombocytopenia [2], [9], [10]. Pathophysiology of hematological involvement in GD have been related to hypersplenism and hematopoietic impairment due to BM infiltration by Gaucher cells, but an intrinsic hematopoietic stem/progenitors cells (HSPCs) defect linked with GBA deficiency cannot be ruled out.

Little is known regarding the functional integrity of GD patients BM microenvironment. Since BM mesenchymal stem cells (MSCs) are multipotent progenitors able to differentiate along various mesenchymal cell subtypes *in vitro* and *in vivo* and particularly into osteoblasts, we hypothesized that skeletal symptoms observed in GD may be, at least in part, the consequence of MSCs intrinsic abnormalities [11]. MSCs also support hematopoiesis through extracellular matrix proteins expression and cytokines secretion. Therefore, hematological symptoms observed in GD may be the indirect consequence of MSCs abnormalities or/and HSPCs intrinsic alteration [12].

We actually previously described the role of GBA deficiency on MSCs and HSPCs functions in an *in vitro* chemical model of GD induced by conduritol β epoxide (CBE) treatment, which leads to a residual GBA activity of 1% compared to untreated cells and impaires stem cells function [13], [14]. In a recent study, Campeau et al reported, in a type 1 GD patient, that MSCs displayed a low GBA activity and accumulated glucocerebroside [15]. MSCs phenotype and differentiation capacities were found to be normal but an altered inflammatory secretome was observed. COX2 (cyclooxygenase-2), IL8 (Interleukin-8), PGE2 (Prostaglandin-E2) and CCL2 (Chemokine ligand-2) levels were increased compared to healthy donors MSCs, thus suggesting a contribution of MSCs in skeletal disease. These data have been recently confirmed in a mouse model of GD which displayed skeletal manifestations partly related to an impairment of BM MSCs properties [16].

In this prospective study, we analyzed MSCs and HSPCs functionality in a series of 10 type 1 GD patients.

## Methods

### Ethics statement

BM and blood samples were obtained from 10 type 1 GD patients after informed consent according to French authorities' guidelines (ANSM, Agence Nationale de Sécurité du Médicament et des produits de Santé; clinical trial agreement #A90674-60 and registered at www.clinicaltrials.gov as NCT01439607). French authorities' specifically approved this study and patients provided their written consent to participate. Healthy donors' blood as well as BM samples were obtained after informed consent according to approved institutional guidelines (Assistance Publique-Hôpitaux de Paris, Paris, France).

Animal work: mice were housed in pathogen-free animal facility under conditions approved by the Animal Care and Use committee of the Institut Universitaire d'Hématologie, Paris, France that also approved this study. Mice were weekly examined for any signs of sickness, such as prostration, severe weight loss, all efforts were made to minimize suffering.

### GBA activity measurement

GBA activity was measured in monocytes, lymphocytes, granulocytes, CD34^+^ cells and MSCs with PFB-FDGlu (5′-pentafluorobenzoylaminofluorescein-di-β-D-glucoside) as substrate. Fluorescence was measured on the FL-1 emission channel by flow cytometry as previously described [16], [17], [18]. Results were expressed as an index defined as the ratio between median fluorescence intensity (MFI) of cells incubated with PFB-FDGlu alone and median fluorescence intensity of cells incubated with CBE and PFB-FDGlu.

### *In vitro* HSPCs studies

BM-CD34^+^ cells were isolated using the direct CD34 Progenitor Cell Isolation Kit (Miltenyi Biotec, Paris, France). Enumeration of BM-CD34^+^ cells from GD and controls was performed using the Stem-Kit reagents according to manufacturer's protocol (Beckman Coulter, Roissy, France).

For CFUs assays, CD34^+^ cells were seeded in Methocult H4434 (Stem Cell Technologies, Grenoble, France) as previously described [13].

CD34^+^ cells were *ex vivo* expanded in IMDM medium supplemented with 10% fetal bovine serum, SCF (50 ng/mL), TPO (50 ng/mL), IL-3 (20 ng/mL) and Flt3-L (20 ng/mL) (Tebu-bio, Le Perray en Yvelines Cedex, France) during 12 days. Cells were counted at day 6 and 12.

Long-Term Culture Initiating-Cell (LTC-IC) were performed as previously described [19]. Briefly, 400 CD34^+^ from GD patients or controls were plated onto healthy donors irradiated mesenchymal stem cells (MSCs) and incubated for 5 weeks. Weekly, the entire content of triplicate was harvested and plated in methylcellulose medium (Stem Cell Technologies). Colonies were counted at day 14.

Hematopoietic supportive capacity of MSCs derived from patients or healthy donors was evaluated by using limiting dilution assays for determination of LTC-IC frequency after 5 weeks, according to the Poisson statistical model (L-Calc™, StemCell Technologies).

### NSG mice

Four- to 8-week-old NSG mice [20] (Jackson Laboratory, Bar Harbor, MI) were sublethaly irradiated (2,25 Gy) before transplantation by retro-orbital injection of 1.10^5^ CD34^+^ cells. Mice were anesthetized with 2,2,2-Tribromoethanol (Sigma-Aldrich) before intra femoral BM aspirate 7 weeks after transplantation.

### MSCs culture and characterization

BM MSCs were isolated and expanded as previously described in Minimum Essential Medium-α (Invitrogen, Cergy Pontoise, France), supplemented with 10% Fetal Bovine Serum (HyClone, Logan, UT), Glutamax-I (2 mM; Invitrogen), bFGF (1 ng/mL; R&D Systems, Lille, France) and antibiotic/antimycotic (1%, Invitrogen) [21]. Monoclonal antibodies conjugated with either fluoresceine isothiocyanate or phycoerythrin and directed to CD34, CD45, CD73, CD90, CD13, CD29, CD105 or matched isotype control (all purchased from Becton Dickinson, Le Pont de Claix, France) were used for immunophenotyping. Data were acquired and analyzed on a five-parameter flow cytometer (FACScalibur, Becton Dickinson, San Jose, CA) with CellQuestPro software (Becton Dickinson).

### CFU-F quantification

BM mononuclear cells were seeded at 1.10^4^ cells/cm^2^ in complete medium. At day 14, colony forming unit-fibroblasts (CFU-F) were fixed with methanol and stained with Giemsa. Colonies were quantified by microscopic examination.

### Long term proliferation assays

MSCs were plated in triplicates in 6 well plates at the initial density of 5.10^3^ cells/cm^2^. Population doubling per passage was calculated as previously described [16].

### Cell cycle analysis

Cells cycle analysis was assessed on MSCs at passage 3 to 4. Cells were trypsinized, fixed with 70% ethanol and stored for at least 24 h at −20°C. After ethanol removal, cells were washed twice with PBS and were incubated with staining solution (1 mg/mL RNase A (Roche Applied Science, Meylan, France) with 10 μg/mL propidium iodide (Sigma Aldrich) in PBS. Cell cycle analysis was performed by flow cytometry, and proportion of cells in the G0/G1, S and G2/M phase were calculated using Cell Quest Pro software (BD Biosciences).

### Microtubules and nuclear fluorescent staining

For immunofluorescent staining, cells at passage 3 to 4 were grown on coverglass coated with fibronectin (1 mg/mL, Calbiochem, Meudon, France). After adhesion, cells were fixed for 15 min in 4% paraformaldehyde, permeabilized for 3 min with 0.1% Triton in PBS and washed three times in PBS. Cells were incubated with 3% BSA in PBS to block unspecific binding of the antibodies. Microtubules were labelled with rat anti-alpha tubulin antibodies (1/500, 30 min, Ab Serotec, Düsseldorf, Germany) revealed with goat anti-rat Cy3 (1/500, 45 min, Interchim, Montluçon, France). Hoechst (0.5 µg/mL) was used for nuclear staining. Slides were then mounted with Mowiol solution and observed using a Nikon fluorescence microscope. Nucleus size distribution was measured with the Image J software.

### RT-qPCR

Total RNA was extracted (RNeasy mini kit, Qiagen, Courtaboeuf, France) and cDNA was prepared with the High Capacity cDNA Reverse Transcription kit following the manufacturer's instructions (Applied Biosystems, Courtaboeuf, France). *HMBS* hydroxymethylbilane synthase) was used to normalize expression data and the 2^–ΔΔCT^ method was applied. Final results were expressed as the *n*-fold differences in target gene expression in GD MSCs compared with control MSCs. PCR were performed with Taqman gene expression inventoried probes (*Runx2*: Hs00231692_m1, *HMBS*: Hs00609297_m1) on 7900 real time PCR system (Applied Biosystems).

### MSCs Osteogenic differentiation

For osteogenic differentiation, 70–80% confluent cultures at passage 4 to 5 were incubated in 6 wells micro-plates in osteogenic medium consisting of Dubelcco's modified Eagle medium (DMEM, Invitrogen) with 4.5 g/L glucose supplemented with 10% FBS, 10^−7^M dexamethasone, 50 µg/mL ascorbic acid and 3 mM inorganic phosphate (for alizarin red staining).

Alkaline phosphatase (ALP) activity was determined by the colorimetric conversion of p-nitrophenol from p-nitrophenylphosphate and normalized to total protein (BCA, Pierce) as previously described [16]. Results were expressed as nmol p-nitrophenol/min/mg protein.

### Osteoclast culture and bone resorption quantification

Peripheral blood mononuclear cells (PBMCs) were isolated by Ficoll gradient density and monocytes cells were purified using CD14 microbeads according to the manufacturer's protocol (Miltenyi Biotec, Paris, France). Monocytes were plated on BD Biocoat Osteologic MultiTest Slides (BD Biosciences) at 8.10^4^ cells/well in Minimum Essential Medium-α supplemented with 10% Fetal Bovine Serum, 1% antibiotic/antimycotic, 100 ng/mL M-CSF (monocyte colony stimulating factor), 100 ng/mL RANK-L (Receptor Activator for Nuclear Factor κ B Ligand) and 25 ng/mL TNF-α (Tumor Necrosis Factor alpha). Cells were fed twice weekly and at day 14, resorption pits were quantified using Von Kossa staining. Briefly, slides were fixed with 4% PFA and stained with 5% silver nitrate solution (Sigma Aldrich) for 30 minutes. Staining development was performed with 5% thiosulfate sodium (Sigma Aldrich). Wells were photographed and analyzed as percentage of resorbed areas according to the Technical Bulletin #444 from BD Biosciences.

In studies examining the effect of conditioned media (CM) from controls and GD patients MSCs, CM were harvested after 72 hours and kept frozen until resorption assay. After CD14^+^ purification, 8.10^4^ monocytes were cultured with 2X osteoclastic medium supplemented with CM from GD or control MSCs (1∶1, volume/volume). CM was added at each media change.

### Soluble factors production

MSCs supernatants from control and GD were used to quantitatively analyzed Dickkopf-related protein-1 (DKK-1), Stromal cell-derived factor-1 (SDF-1), osteopontin (OPN), osteoprotegerin (OPG), monocytes chemotactic protein-1 (MCP-1), interleukin-6 (IL-6) and interleukin-8 (IL-8) levels. Analyzes were performed on MSCs at passage 3 to 4 and supernatants were harvested 72 hours after trypsinisation. All testing were performed by enzyme-linked immunosorbent assay (ELISA) using commercially available kits (R&D Systems) according to the manufacturer's instructions. Quantitative analysis of PGE2 was performed using a competitive binding technique according to the manufacturer's protocol (R&D systems).

### Statistical analysis and results description

Differences between values were evaluated by Student's t-tests. When normality tests or equal variance tests failed, Mann-Whitney tests were performed. A value of *P*<0.05 was used to define statistical significance.

The box plots summarize the following statistical measures: medians are represented as horizontal bars (−), mean as plus (+) upper and lower quartiles are represented on the top and the bottom of the box, minimum and maximum data values are on the top and the bottom of the whiskers represented as dash (−) and atypical values are shown as circle (○). Number mentioned in results section represents median.

## Results

### Patients' characteristics and GBA activity

Characteristics of the 10 GD patients prospectively included in this study are presented in Table 1. GD was confirmed by genotype analysis and by the detection of the enzymatic deficiency. Median age was 59.5 years (range 32–77 years). Severity scores presented in Table 1 are at study entry. Five patients displayed hematological disorders, four had monoclonal gammopathies, and bone abnormalities were observed in all patients. Seven patients received enzyme replacement therapy at the time of inclusion. None of the included patient was treated with B12 vitamin, and only one received iron therapy.

**Table 1:** GD patients characteristics.

Patient n°	Genotype	Year, and age at diagnosis	Splene ctomy	SMG	HMG	Hb (g/dl)	MCV (fl)	Platelet (×103/L)	WBC/ mm^3^	Chitotriosidase activity (nmol/h/ml)	Ferritinemia (µg/l)	Gamma- globulinemia (g/L)	Bone pain	Radiological manifestations (Xrays, MRI)	ERT (Year and age of start)	Time between last ERT and inclusion (days)
1	N370S/R496P	1983 (24)	+	splenectomy	+	13,2	91	351	6200	NA	112	13,9	+	pelvis infarct	1997 (38)	335
2	N370S/R463C	1990 (12)	−	−	−	13	92,4	202	7810	280	14	11,6	+	hip osteonecrosis, femurs and tibia infarct	1997 (19)	2
3	N370S/R120W	1981 (34)	−	+	−	13,6	87	104	4400	24 000	1501	13,4	−	femurs, tibia BI, asymptomatic femurs, tibia infarct	no treatment	no treatment
4	N370S/L324P	1992 (57)	−	+	+	13	NA	51	2900	3930	1030	16,8 (monoclonal gammopathy)	−	Femur, tibia infarct	2009 (74)	10
5	N370S/L324P	1994 (61)	−	+	+	11,5	94	77	3900	41 500	2235	10,4	−	right hip prothesis, left osteonecrosis	No treatment	no treatment
6	N370S/W494X	1997 (48)	+	splenectomy	−	14,9	96	369	7100	Deficit	579	8,4	+	Humerus, pelvis infarct	2005 (56)	7
7	N370S/G202R	1984 (34)	−	−	−	12,7	93	174	4400	23 300	331	16,9 (monoclonal gammopathy)	+	hip prothesis, shoulder prothesis	2002 (52)	260
8	N370S/R120W	1969 (18)	+	splenectomy	−	13	92	303	7070	4900	NA	NA	+	humerus infarct, BI	2002 (51)	294
9	N370S/L444P	2010 (53)	−	+	+	14,7	88	130	6400	13 200	807	13,4 (monoclonal gammopathy)	−	femurs infarct	no treatment	no treatment
10	N370S/D218A	1998 (33)	−	+	−	16	85	127	5030	1700	307	7,5 (monoclonal gammopathy)	−	femurs, tibia BI	04/01/2010	8

SMG, splenomegaly; HMG, hepatomegaly; Hb, hemoglobin; MCV, mean corpuscular volume; WBC, white blood cell; MRI, magnetic resonance imaging; BI, bone infiltration; ERT, enzyme replacement therapy.

**Figure 1:**
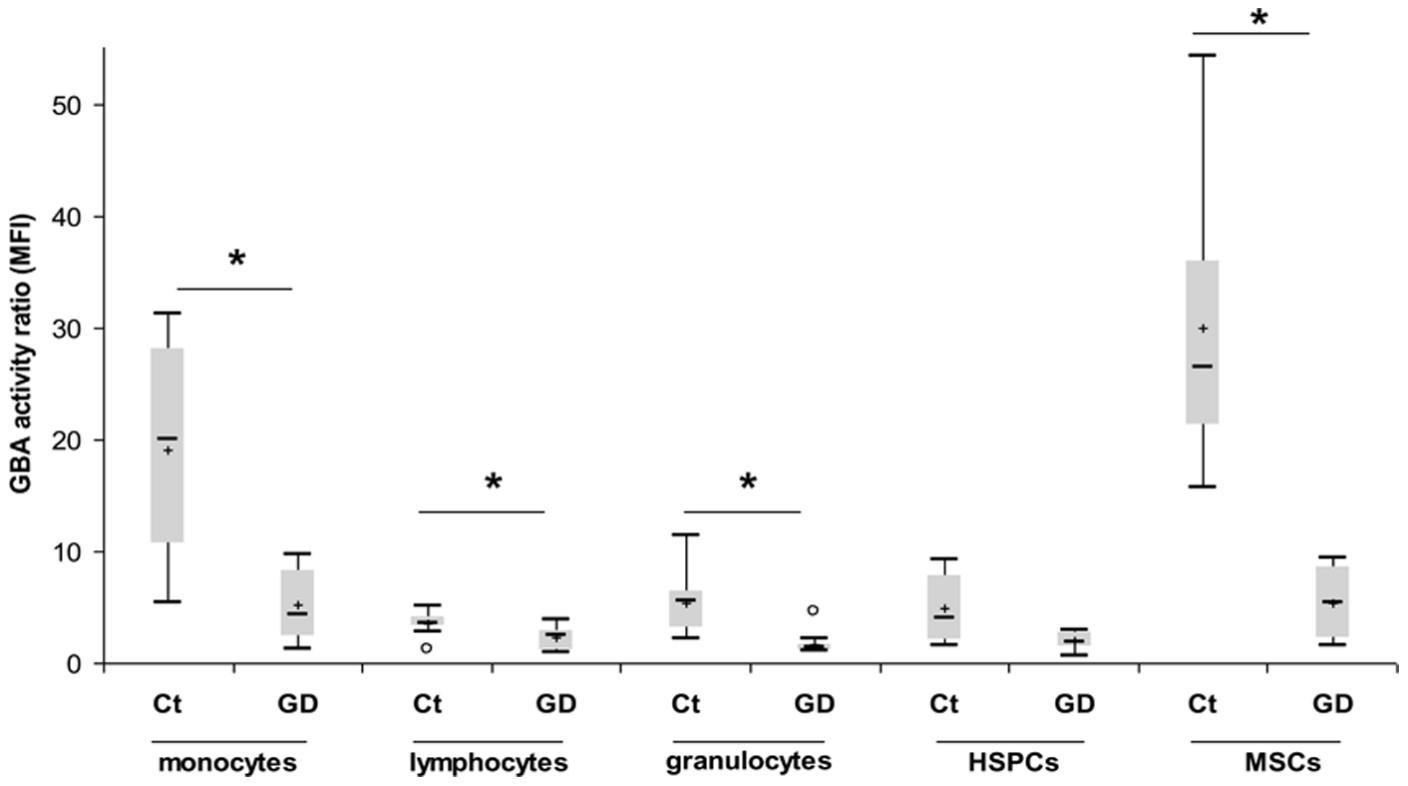
GBA activity measurement in blood and BM cells. GBA activity was measured in blood and BM cell subtypes of GD patients (GD) and healthy donors (Ct). Results are expressed as an index defined by the ratio between median fluorescence intensity (MFI) of cells incubated with PFB-FDGlu alone and MFI of cells incubated with CBE and PFB-FDGlu (blood: n = 10 for GD and controls; BM: n = 8 for GD and 6 for controls).

GBA activity was measured into sub-cellular fractions of blood, in BM-MSCs and in BM-CD34^+^ cells. Flow cytometry was chosen to assess GBA activity since it allows GBA activity measurement in sample cell subpopulation. Compared to controls, GD patients GBA activity was significantly decreased in all blood and BM cell sub-type (Figure 1). Various degrees of GBA deficiency was observed among cell population: mean GBA residual activity in GD patients monocytes was of 28% compared to controls (19% and 30% in ERT treated and untreated patients, respectively) and of 39% in HSPCs (36% and 41% in ERT treated and untreated patients, respectively). Interestingly, we observed that MSCs displayed the higher GBA activity among all cell subtype. Furthermore, GD patients MSCs were also found to have the highest GBA deficiency (21% of residual activity, 20% and 23% in ERT treated and untreated patients, respectively).

### Hematopoiesis in GD patients

**Figure 2:**
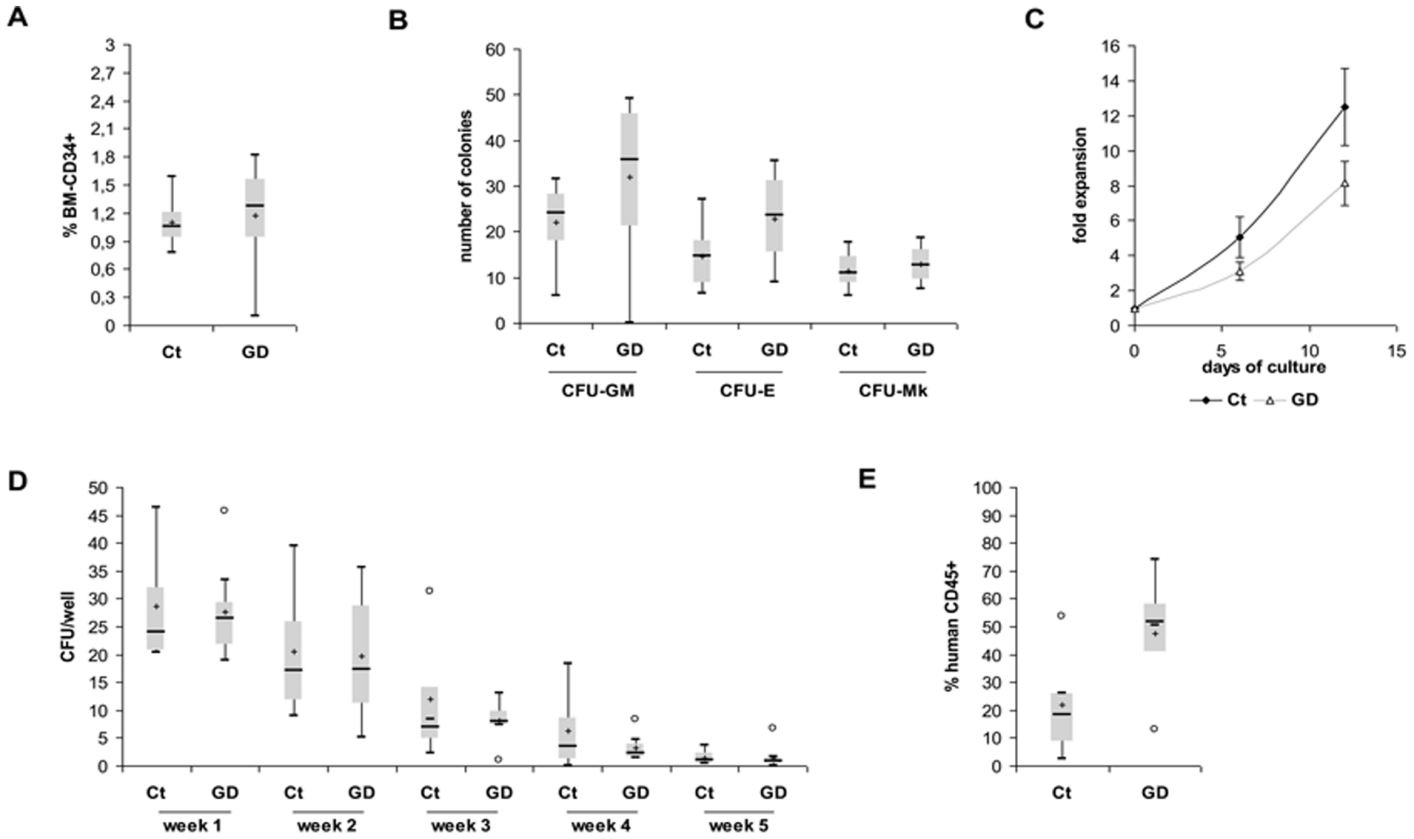
Hematopoiesis in GD. (A) Relative number of CD34^+^ cells was measured in GD (n = 7) and controls (n = 10) BM samples according to ISHAGE Guidelines [34]. (B) CD34^+^ cells clonogenic potential. Granulocyte–macrophage colony-forming units (CFU-GM), erythroid burst-forming units (BFU-E), and megakaryocyte colony-forming units (CFU-Mk) were scored at day 14 by direct visualization using an inverted microscope (n = 8 and 10 for GD patients and controls, respectively). (C) BM-CD34^+^ cells *ex-vivo* expansion. Total nucleated cell (TNCs) number was determined after 6 and 12 days culture (n = 6 and 10 for GD and controls respectively). Results are presented as TNCs fold expansion. (D) Primitive hematopoiesis was evaluated by LTC-IC assay. GD patients and controls CD34^+^ cells were cultured on healthy donors MSCs during a period of 5 weeks. All experiments were performed in triplicate (n = 4 and 8 for GD patients and controls, respectively). (E) Four- to 8-week-old NSG mice were transplanted by retro-orbital injection with 1.10^5^ BM CD34^+^ cells from GD patients or controls. The presence of human CD45^+^ cells was assessed by flow cytometry 7 weeks later (n = 5 mice engrafted with CD34^+^ cells from 2 healthy donors and 4 mice engrafted with CD34^+^ cells from 2 GD patients).

In order to study the impact of GBA deficiency on GD HSPCs, CD34^+^ cells were isolated from patients BM and compared with those of healthy donors. Consequences of GBA deficiency were evaluated on mature and primitive hematopoiesis. The percentage of BM-CD34^+^ cells was similar in GD patients and healthy donors (1.10% and 1.17% respectively, *P* = 0.773; Figure 2A). We next analyzed the CD34^+^ differentiation capacities and found that the median number of CFU-GM, BFU-E and CFU-Mk were similar between patients and controls (CFU-GM: 35.75 versus 24; BFU-E: 23.5 versus 14.75; CFU-Mk: 12.75 versus 11; *P* = 0.148, 0.068 and 0.475 respectively; Figure 2B). CD34^+^ cells, isolated from both GD and healthy donor BM, were then *ex-vivo* expanded during 12 days in a SCF/TPO/IL-3/Flt3-L containing medium. The nucleated cells fold expansion was comparable in the two groups (mean +/− SEM: 8.14+/−1.27 versus 12.47+/−1.49 at day 12 respectively, *P* = 0.9; Figure 2C), and no difference was observed regarding the phenotype of expanded cells (data not shown).

We also evaluated hematopoiesis in long-term culture and found that the number of colony-forming cells (CFCs) obtained weekly over a period of 5 weeks did not differ significantly between GD patients and controls (Figure 2D; *P*>0.05), thus suggesting that *in vitro* CD34^+^ functionality was not impaired in the tested patients.

To determine the potential of HSPCs to restore hematopoiesis *in vivo*, GD and controls CD34^+^ cells were tested in xenograft experiments by injection into NSG mice. Engraftment was analyzed 7 weeks after transplantation. Relative number of human CD45 positive BM cells was comparable between GD and healthy donors (Figure 2E; *P* = 0.129). Engraftment of NSG mice with GD or controls CD34^+^ cells generated both B lymphoid and myeloid lineages as shown by the presence of CD19^+^ and CD33^+^ cells (Figure S1). CD34^+^ cells were also found in mice BM (0.79+/−0.16% for GD and 1.85+/−0.14% for controls, *P* = 0.052). *In vivo* experiments thus confirmed that GBA deficiency in GD CD34^+^ did not significantly modify engraftment capacities.

Because BM microenvironment regulates HSPCs biology, we aimed to study the potential role of MSCs on HSPCs functions. Healthy donors CD34^+^ cells were cultured on GD or healthy donors MSCs. Interestingly, we found that CFCs frequency was significantly decreased when GD-MSCs were used (1/890 (ranging from 1/943 to 1/600) versus 1/403 (ranging from 1/424 to 1/314) for control group, *P* = 0.023). Thus, GD-MSCs displayed lower hematopoietic supportive capacity than control-MSCs.

### GBA deficiency impaired MSCs characteristics

**Figure 3:**
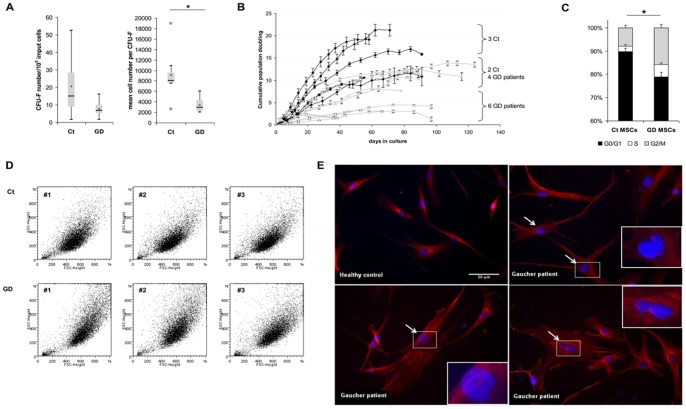
GD-MSCs characterization. (A) Histograms represents CFU-F quantification per 10^5^ mononuclear cells (left panel) and mean cell number per CFU-F (right panel) obtained after culture of control (Ct) or GD BM samples (n = 10 and 7 for GD patients and controls respectively). (B) MSCs growth kinetics: cumulative population doubling (CPD) are shown for Ct and GD-MSCs (n = 5 and 10 respectively). (C) Cell cycle analysis was performed by flow cytometry on Ct and GD-MSCs. The percentage of cells in each phase of the cell cycle was determined by gating G0/G1, S and G2/M cells (n = 8 and 6 respectively). (D) Representative FSC/SSC flow cytometry profile of Ct and GD-MSCs. (E) Ct and GD-MSCs were stained with anti-alpha-tubulin (red) and with Hoechst for nuclear staining (blue). Typical non-round nuclei (angled panels) and large nuclei (arrows) were observed in GD-MSCs. Scale bars represent 50 µm. A and C: * indicates *P*<0.05.

MSCs progenitors from GD were analyzed for their ability to form fibroblastic colonies (CFU-F). CFU-F frequency did not significantly differ between GD and healthy donors cells (7 and 15 CFU-F respectively, *P* = 0.118, Fig. 3A). However, mean cell number per CFU-F was decreased in GD compared to healthy donors (2893 and 8000 cells/CFU-F respectively, *P* = 0.004).

The growth potential of MSCs from 10 GD patients was evaluated over the time and compared to controls (Ct, Figure 3B). Cell proliferation was heterogeneous for both GD and controls. However, MSCs from 6 GD patients (patients #4, 5, 7, 8, 9 and 10) had significantly lower proliferative capacity than control MSCs. Growth potential of MSCs from 4 other GD patients (patients #1, 2, 3 and 6) was similar to that of 2 controls. Lastly, none GD-MSCs proliferation reached the one observed for 3 controls. No correlation between clinical or biological patients characteristics and cell proliferation decrease could be found to explain the heterogeneous data obtained with GD-MSCs. GD-MSCs proliferation impairment was not related to an increased apoptosis or senescence (Figure S2A and B). MSCs phenotype was also similar between GD and controls (data not shown).

Cell cycle analysis was performed in order to investigate the abnormal expansion capacities of GD-MSCs. The relative number of cells in G0/G1 was significantly decreased in GD compared to healthy donors (78.9 and 89.6 respectively, *P* = 0.001, Figure 3C) whereas the G2/M-phase was increased (15.8 and 7.8 respectively, *P* = 0.001). These data suggest that abnormal proliferation observed in GD-MSCs was related to cell cycle alteration.

The morphology of GD-MSCs was also altered as shown by forward scatter/side scatter (FSC/SSC) flow cytometric profile from 3 controls and 3 GD patients (Figure 3D). GD-MSCs volume (FSC) and cytoplasmic granularity (SSC) were increased, suggesting that glucosylceramide accumulation modified MSCs morphology. Moreover, immunofluorescent staining of cytoskeleton (microtubules) and nucleus (Hoechst) revealed the presence of 10% of cells with abnormal nuclear shape (non-round nuclei) among MSCs from GD whereas it was never observed in healthy donors MSCs (Figure 3E). We also found that nuclear size was increased in GD-MSCs as compared to healthy donors (278+/−97 µm^2^ and 175+/−49 µm^2^, respectively, *P* = 0.002, n>150 cells from 3 healthy donors and 6 GD patients).

### Bone remodeling balance in GD

Bone mass abnormalities observed in GD may be the consequence of an unbalance between bone formation by osteoblasts derived from MSCs, and bone resorption by osteoclasts of hematopoietic origin.

**Figure 4:**
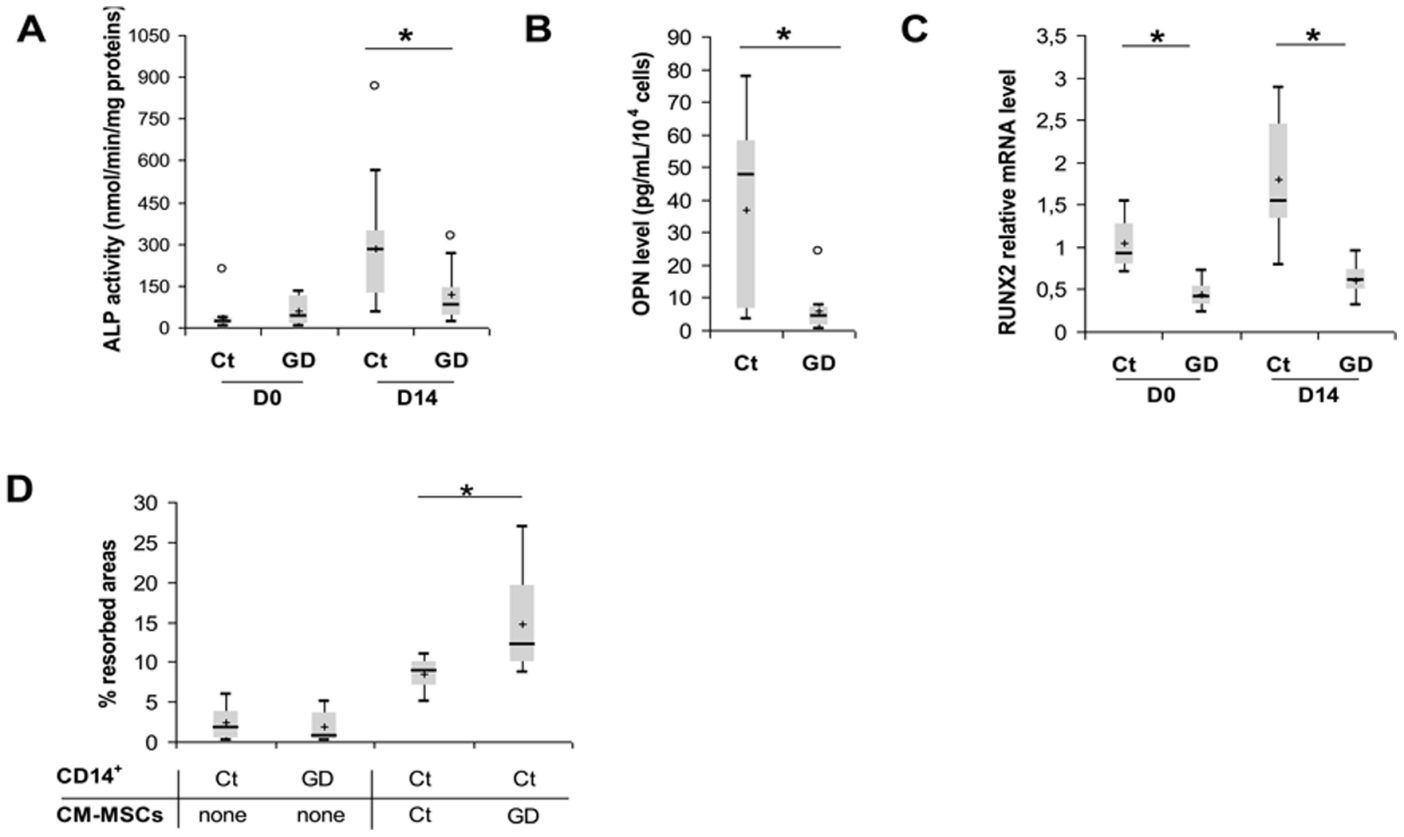
Osteoblastic and osteoclastic balance in GD. *MSCs osteogenic differentiation:* (A) Box plots represent ALP activity at D0 and after 14 days of differentiation for Ct and GD. Results are expressed as nmol/min/mg protein (n = 10). (B) Osteopontin levels were measured by ELISA at D0 and D14 after differentiation in culture supernatants of Ct and GD-MSCs (n = 10). (C) Relative expression of osteogenic transcription factor *RUNX2* measured by RTqPCR at D0 and D14 after differentiation (n = 10). (D) Resorption activity of CD14^+^-derived osteoclasts cultured on biocoat bone matrix in osteoclastic media: Quantification of resorption potential of monocytes-derived osteoclasts from Ct or GD after 14 days of differentiation or from Ct-osteoclasts in the presence of Ct or GD treated MSCs conditioned media (CM) (n = 6).

The *in vitro* potential of MSCs to differentiate along the osteoblastic lineage was evaluated in GD-MSCs and compared to control MSCs. Phosphatase alkaline (ALP) activity was measured in basal condition, *i.e.* in growth medium (D0), and after 14 days in osteogenic medium. At D0, no statistical difference was observed between GD and Ct (40.83 and 21.41 nmol/min/mg proteins respectively, *P* = 0.302, Figure 4A). However, ALP activity was significantly decreased at D14 in GD-MSCs-derived osteoblasts compared to control (78.59 and 280.1 nmol/min/mg proteins respectively, *P* = 0.037, Figure 4A). Because osteopontin (OPN) has been used as a marker of osteoblast function, we measured OPN level in supernatant after 14 days of culture in osteoblastic medium [22]. Similarly to ALP activity, OPN secretion in MSCs-derived osteoblasts was significantly decreased in GD compared to healthy donors (6.02 and 37.11 pg/mL/10^4^ cells respectively, *P* = 0.017, Figure 4B). In addition, the expression of *RUNX2*, the key transcription factor involved in osteogenic differentiation, was decreased in GD-MSCs compared to controls, at D0 and D14 (0.41 versus 0.92 respectively, *P* = 0.001 at D0; 0.63 versus 1.79 respectively, *P* = 0.001 at D14, Figure 4C). Taken together, these data revealed an impaired MSCs differentiation capacity in GD, suggesting that bone abnormalities observed in patients may be the consequence of a MSCs intrinsic defect.

*In vivo*, bone mass is also regulated by osteoclasts that induce bone resorption. In order to evaluate osteoclasts function in GD, monocytes were cultured in osteoclastic medium during 14 days on calcium phosphate matrix and bone resorption areas were measured. No significant difference was observed between bone resorption areas from osteoclasts obtained after differentiation from GD or healthy donors CD14^+^ cells (0.725 and 1.821% of resorbed areas respectively, *P* = 0.937, Fig. 4D, left panel). However, resorption areas were increased when monocyte-derived osteoclasts were cultured in the presence of GD-MSCs conditioned media compared to controls (12.1 and 8.8% of resorbed areas respectively, *P* = 0.030, Figure 4D, right panel). These data suggest that GD-MSCs secrete soluble factors that induce osteoclastic differentiation or activation. Bone mass modification observed during GD could thus be a consequence of MSCs intrinsic abnormalities, rather than a direct impairment of osteoclasts.

### MSCs secretome

**Figure 5:**
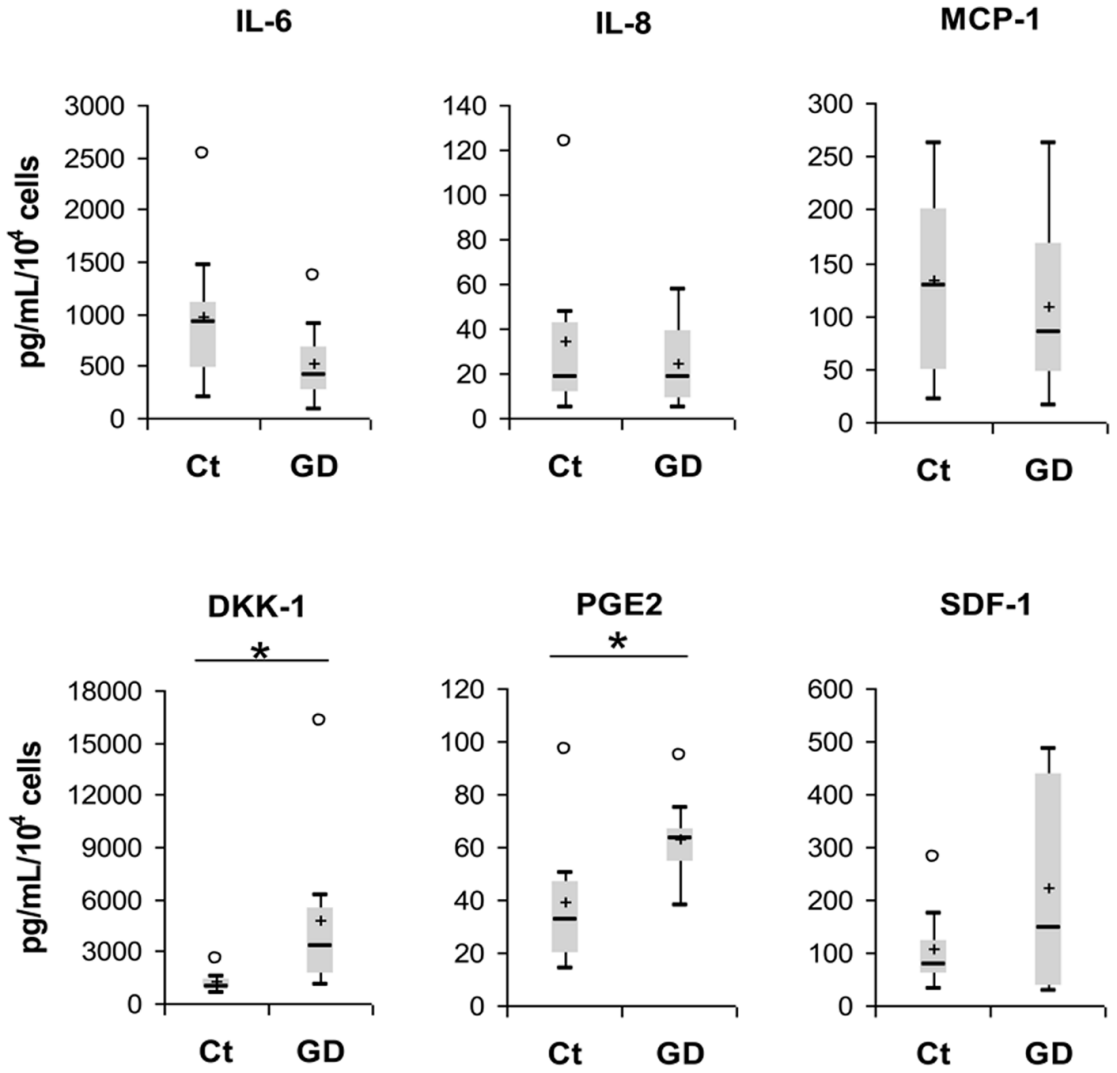
MSCs' soluble factors secretion. Interleukin-6 (IL-6), IL-8, Monocytes chemotactic protein-1 (MCP-1), Dickkopf-related protein-1 (DKK-1), prostaglandin-E2 (PGE2), and stromal cell-derived factor-1 (SDF-1) levels were measured in supernatants obtained from controls and GD-MSCs (n = 8). Levels were normalized to the number of cells.

Alterations of osteoclasts function and of long-term hematopoiesis support described above in GD cells are likely related to MSCs deficiency. Mechanisms underlying this phenomenon may include secretome abnormalities. Campeau et al. reported that MSCs from one patient with type 1 GD had an altered secretome [15]. We also reported similar results in the *in vitro* chemical model of GD, induced with CBE [14]. MSCs secretome was explored in 8 patients of our cohort (Figure 5).

Consistently with our previous study on MSCs treated with CBE, we found that MSCs from GD secrete significantly more Dickkopf-related protein-1 (DKK-1) than healthy donors (3226 and 976 pg/mL/10^4^ cells respectively, *P* = 0.020). Moreover, prostaglandin-E2 (PGE2) level was also increased in GD-MSCs supernatant (63.00 and 32.65 pg/mL/10^4^ cells respectively, *P* = 0.038). However, Monocytes chemotactic protein-1 (MCP-1) and Interleukin-8 (IL-8) levels, that were found to be increased in CBE treated MSCs and in Campeau's study, were found similar between GD and controls (83.83 and 127.08 pg/mL/10^4^ cells respectively for MCP-1, *P* = 0.577; 17.90 and 18.19 pg/mL/10^4^ cells respectively for IL-8, *P* = 0.525). IL-6 and stromal cell-derived factor-1 (SDF-1) levels were also similar between MSCs from GD and controls (418.62 and 918.37 pg/mL/10^4^ cells respectively for IL-6, *P* = 0.161; 144.41 and 77.43 pg/mL/10^4^ cells respectively for SDF-1, *P* = 0.798).

## Discussion

Mechanisms underlying bone abnormalities and hematological manifestation observed in GD are still a matter of debate. We hypothesized that GBA deficiency may be responsible for a deregulation of MSCs and/or HSPCs functional properties. Actually, MSCs are precursors of several cellular lineage including osteoblasts. They also play a central role in the BM niche by supporting HSPCs maintenance through the secretion of soluble factors and cell-cell interactions [11], [12]. Thus, hematological manifestation in GD may be the consequence of an intrinsic abnormality or of an altered interaction with components of the BM niche.

We have previously shown, in a chemical model of GD obtained with CBE treatment, that GBA deficiency in MSCs lead to an increased secretion of several soluble factors that regulate bone metabolism and to an increased activation of resorbing osteoclasts [14]. Moreover, in the same *in vitro* model, GBA deficiency in HSPCs impairs mature and primitive hematopoiesis [13]. In order to evaluate the consequences of GBA deficiency in patients BM cells, we designed a clinical trial in which 10 type 1 GD patients were prospectively included, and MSCs/HSPCs studied.

We first evaluated GBA activity in sub-cellular fractions from blood and BM using a flow cytometric approach. Interestingly, we found the highest GBA deficiency in MSCs with 21% of residual activity, suggesting that MSCs may be a key component of abnormalities observed in GD.

In our previous *in vitro* studies, cells were treated by 0.5 mM CBE. Such treatment induces a reduction of GBA activity to 1% of untreated cells activity, in both MSCs and in HSPCs [13], [14]. In contrast with this chemical model, data collected in patients argues for a conserved *in vitro* and *in vivo* HSPCs function. These discrepancies may be explained by the GBA residual activity. The “critical threshold” hypothesis, proposed by Conzelmann and Sandhoff, was actually demonstrated in fibroblasts from patients with β-hexosaminidase-A or arylsulfatase-A deficiency and in the J774 murine macrophage cell line treated by CBE [23], [24], [25]. In these models, it has been shown that an increased storage occurred when enzyme activity was reduced to 10-15% of the normal control levels. We analyzed HSPCs function in a group of 10 patients. Because of the rarity of the disease and of the prospective inclusion of the patients, our cohort was heterogeneous and only one patient displayed severe hematological disorders. Moreover, some patients were splenectomized, but we did not find correlation between splenectomy and *in vitro* results. The residual GBA activity observed in HSPCs patients (39% of controls, range 13 to 61%) may thus be sufficient to ensure HSPCs normal function. However, an intrinsic HSPCs abnormality in patients with lower GBA activity cannot be ruled out.

The hematopoietic functional deficit we observed after long-term culture of CD34^+^ cells on GD-MSCs suggests that microenvironment may contribute to the development of hematopoietic abnormalities in GD, consistently with previous studies [14], [15]. Moreover, GBA residual activity in MSCs was found to be similar to the values determined in the “critical threshold” hypothesis.

The first consequence of GBA deficiency in GD-MSCs was the impairment of proliferation in short-term culture (as shown by mean cell number per CFU-F decrease) and over the time. Inhibition of proliferation was related to an increase in the G2/M phase of the cell cycle. Similarly to the CBE *in vitro* model, GD-MSCs were found to be bigger and exhibited abnormal shape and nucleus size. Lipids accumulation, and modification of the lipid rafts composition, have already been observed in CBE-treated macrophage and in fibroblasts from type 1 and 2 GD [26]. Lipid rafts are known to play a key role in cellular functions such as proliferation, apoptosis, adhesion, signal transduction and membrane trafficking and cytokinesis [27]. These data suggest that alteration of lipid metabolism in GD-MSCs may induce cytokinesis defect, cell structure abnormalities and cell proliferation decrease.

Since MSCs are osteoblasts precursors, we studied the ability of GD-MSCs to differentiate along the bone lineage. mRNA level of RUNX2, a key regulator of osteoblasts differentiation, was decreased in GD-MSCs compared to healthy donors. In addition, ALP activity and osteopontin secretion level were found to be decreased in GD-MSCs. Bone mass homeostasis is also dependant on osteoclasts activity. It has been shown, in an vitro CBE chemical model, that conditioned media from CBE-treated PBMC induced ex vivo osteoclastogenesis [28]. We quantified the resorbing potential of CD14+-derived osteoclasts cultured in osteoclastic media on calcium phosphate matrix. Our data revealed that the resorbing potential of CD14+-derived osteoclasts was not significantly different between GD and controls. However, osteoclasts activity was stimulated during culture in the presence of GD-MSCs conditioned media. These data demonstrated that GBA deficiency in GD-MSCs leads to an intrinsic decrease of osteogenic differentiation abilities and to an activation of osteoclasts resorbing activity through soluble factors secretion, thus likely contributing to the abnormal skeletal phenotype observed in GD patients. However, no difference was observed between treated or untreated patients, while it has been reported that ERT treatment may be responsible for inducing osteopenia in some patients [29].

In our previous study, we reported that CBE-treated MSCs secreted increased levels of IL-6, DKK-1, MCP-1, IL-8 and SDF-1. Campeau et al. found that COX2, IL-8, PGE2 and CCL2 levels were increased in a type 1 GD patient. In the present study, we confirmed that GD-MSCs secrete increased levels of DKK-1 and PGE2. DKK-1 overexpression has been shown to correlate with lytic lesions in multiple myeloma and to induce bone loss [30], [31]. PGE2 also stimulate osteoclastogenesis [32], [33]. DKK-1 and PGE2 may thus play a critical role in the development of bone lesions in GD.

These observations were strengthened by the increased resorption we observed when osteoclasts were cultured in the presence of GD-MSCs culture media.

The first type 1 GD mice models were either lethal or do not reproduce the human disease phenotype. More recently, two models, obtained by conditional deletion of the GBA1 gene, have been described. In the one reported by Enquist et al., mice phenotype was characterized by an involvement of hematopoietic organs [34]. The genetic disorder of the hematopoietic system was corrected by a hematopoietic stem cell gene therapy approach [35]. The other mice model associated hepatosplenomegaly, hematologic disease and skeletal complications [16]. Mice MSCs studies revealed that *ex vivo* proliferation was decreased and that osteopenia was related to a reduced osteoblast differentiation. We found similar abnormalities in our GD patient BM cells.

Thus, although the cohort of patients we analyzed was heterogeneous and does not allow to relate the *in vitro* findings with individual patient characteristics, our study provides direct evidence for an impairment of MSCs functions in type 1 GD. That MSCs are defective in type 1 GD opens the possibility of considering this cell type as a key contributor of bone defect observed in patients, and at a lesser extent of hematological manifestation.

## Supporting Information

Figure S1 **Phenotype of engrafted human cells in NSG mice.** Human CD19^+^, CD33^+^, CD3^+^ and CD34^+^ expression was measured in mice BM 7 weeks after transplantation. Histograms represent mean+/− S.E.M.
(TIF)

Figure S2 **Apoptosis and senescence in MSCs from GD.** (A) Flow cytometric detection of cell viability and apoptosis on MSCs from Ct and GD. Apoptotic cells were identified using Annexin-V staining and necrotic cells were identified using 7AAD dye. (B) Representative photographs of senescence assay based on β-galactosidase activity on MSCs from Ct and GD. Senescent cells appeared in blue (magnification ×100).
(TIF)
