# Supplementary figures and images for: A Prospective Study of Bone Marrow Hematopoietic and Mesenchymal Stem Cells in Type 1 Gaucher Disease Patients
Source: PLoS One. 2013 Jul 25;8(7):e69293. doi: 10.1371/journal.pone.0069293 (PMC3723887; doi:10.1371/journal.pone.0069293)

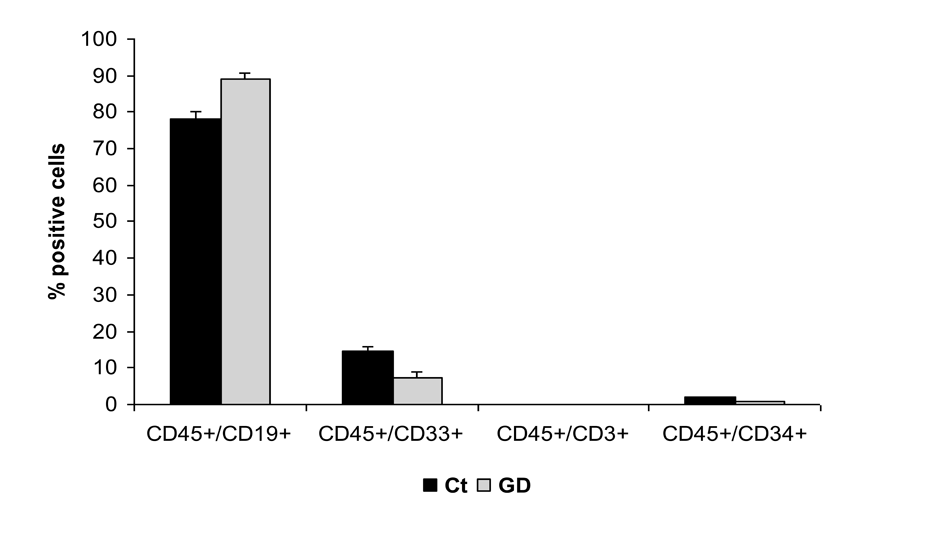

Supplement: Figure S1 — Phenotype of engrafted human cells in NSG mice. Human CD19+, CD33+, CD3+ and CD34+ expression was measured in mice BM 7 weeks after transplantation. Histograms represent mean+/− S.E.M. (TIF) [file pone.0069293.s001.tif]

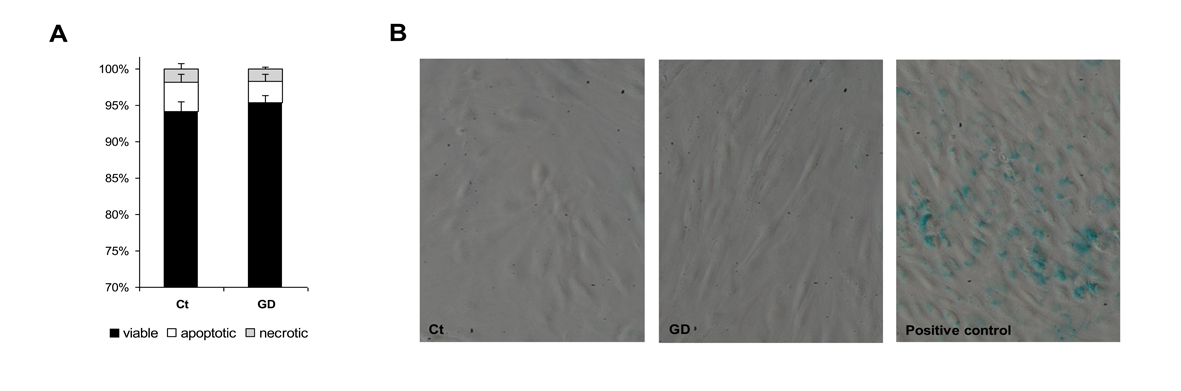

Supplement: Figure S2 — Apoptosis and senescence in MSCs from GD. (A) Flow cytometric detection of cell viability and apoptosis on MSCs from Ct and GD. Apoptotic cells were identified using Annexin-V staining and necrotic cells were identified using 7AAD dye. (B) Representative photographs of senescence assay based on β-galactosidase activity on MSCs from Ct and GD. Senescent cells appeared in blue (magnification ×100). (TIF) [file pone.0069293.s002.tif]
